# Unmasking Atypical Eating Disorders: A Case Series Highlighting Diagnostic Delays and Underrecognized Populations

**DOI:** 10.7759/cureus.110410

**Published:** 2026-06-07

**Authors:** Karan Prasad, Brendan Driscoll, Felicia Gallucci

**Affiliations:** 1 Psychiatry, University of Miami Miller School of Medicine, Miami, USA

**Keywords:** atypical eating disorders, body image disturbances, eating disorders, other specified feeding or eating disorders, underdiagnosis

## Abstract

Atypical eating disorder presentations challenge traditional diagnostic frameworks, leading to underdiagnosis and diagnostic delay, as each patient had prior clinical contacts in which eating disorder pathology went unrecognized. This study examines four cases highlighting unique risk factors, including high-performance athletics, trauma history, substance use, gender dysphoria, and environmental stressors. These patients were not identified as having eating disorders at initial clinical contact, as presenting complaints, including anxiety, alcohol use disorder, attention-deficit/hyperactivity disorder, and environmental stressors, obscured the underlying eating pathology.

Athletes, particularly those in weight-class sports, may engage in restrictive eating while maintaining higher body weight, as seen in Case 1. Trauma history increases vulnerability, yet routine screening remains inadequate. Case 2 demonstrates the interplay between alcohol use disorder and binge-purge cycles, where substance use masked eating pathology. Gender dysphoria in Case 3 contributed to restrictive behaviors as a means of body modification, underscoring the importance of integrating gender-affirming care. Case 4 illustrates how environmental instability can drive disordered eating independent of body image concerns.

These cases emphasize the necessity of broadening screening criteria and provider education to recognize atypical presentations. Addressing coexisting psychiatric conditions and social determinants of health is critical for early intervention and improved outcomes in patients with eating disorders.

## Introduction

Eating disorders involve distorted body image, excessive concern about weight or appearance, and abnormal eating behaviors with significant clinical or psychiatric impact [1]. The most common disorders include anorexia nervosa, bulimia nervosa, and binge eating disorder [1,2]. The Diagnostic and Statistical Manual of Mental Disorders, Fifth Edition (DSM-5) provides criteria for diagnosis, but an increasing number of patients present atypically or with clinical features that diverge from classical diagnostic profiles due to factors such as body weight, comorbid conditions, or alternative motivations for disordered eating behaviors. These atypical presentations can lead to underdiagnosis and gaps in care [3]. This is particularly concerning given the high mortality risk associated with eating disorders; anorexia nervosa has the highest mortality rate among psychiatric conditions (5% mortality within four years of diagnosis) [4]. While eating disorders are commonly associated with adolescents and young adult females, epidemiologic data likely underestimate true prevalence across broader populations, as they disproportionately reflect subgroups with greater healthcare access rather than the full scope of those affected [5]. The National Comorbidity Survey Adolescent Supplement (NCS-A) found a lifetime prevalence of 2.7% in adolescents aged 13 to 18, with females meeting diagnostic criteria more than twice as often as males (3.8% vs. 1.5%) [6]. The median age of onset is 21 years for binge eating disorder and 18 years for anorexia and bulimia nervosa [7]. Key risk factors include body dissatisfaction, depression, low self-esteem, and higher BMI, with additional risks from social media exposure and thin-ideal internalization [8].

Traditional epidemiologic data fail to highlight other at-risk groups. While eating disorders are underdiagnosed in young males due to stigma and under-screening [9], LGBTQIA+ individuals have a six-fold increased prevalence compared to straight males [9,10]. Additionally, diagnostic challenges arise from atypical presentations that extend beyond the Other Specified Feeding or Eating Disorder (OSFED) framework, encompassing a broader range of clinical presentations that fall outside traditional diagnostic expectations [3,10]. The DSM-5 defines atypical anorexia nervosa (AAN) based on significant weight loss, but the lack of standardized diagnostic thresholds across studies creates confusion among providers [11]. Patients with fluctuating weight may also go undiagnosed or be misclassified as in remission despite continued disordered eating behaviors [12]. Given provider misconceptions and unclear diagnostic criteria, improving screening is critical to closing care gaps and ensuring timely treatment [1,10]. This case series presents several atypical cases to illustrate the range of presentations and expand provider awareness. Informed consent was provided by all patients described.

## Case presentation

Case 1

A 17-year-old, single, English-speaking female with obesity and a past psychiatric history of post-traumatic stress disorder (PTSD) presented to an outpatient psychiatry clinic upon referral by her primary care pediatrician with concerns for anxiety and disordered eating. This was her first psychiatric contact. The patient's disordered eating behaviors had been ongoing for several months prior to this evaluation, during which time prior pediatric visits had focused on anxiety and trauma without formal eating disorder screening. The patient reports a childhood history of sexual trauma in her home country and caretaker neglect from those originally responsible for her care when she first immigrated. Guardianship has changed since moving states. She endorses ongoing hypervigilance and avoidance of triggers. When experiencing triggers, she reports adequately mitigating the urge to verbally engage and denies any physical altercations. The patient endorses a distant history of depressed mood and passive suicidal ideation (SI) but denies any history of self-harm or recent history of depressed mood, anhedonia, or SI.

Upon relocating to Florida with a new caregiver, she reports normal sleep, concentration, academic performance, and adequate energy. A large portion of her personal identity is competing in wrestling as an extracurricular activity. She practices six days a week and will often complete her own exercise on days off. Patient reports weighing herself daily to track herself for her wrestling weight class. She denies spending time looking in the mirror. To achieve wrestling-related weight goals, she endorses intentionally restricting her appetite, providing an example of intentional fasting for four days followed by a week of regular eating. Notably, the patient reported feeling she was too thin for her current weight class and expressed a desire to drop to a lower weight class, where she could compete at the higher end of that category, reflecting performance-driven rather than aesthetic motivations for her restrictive behaviors. 

The patient has also reported trialing intermittent fasting to achieve weight loss. After an initial period of patient satisfaction, results began to taper, prompting her to incrementally increase the duration of fasting. She currently reports eating one meal a day with limited water intake, which has resulted in intermittent dizziness upon standing. She denies weakness, malaise, and headaches as well as compensatory behaviors such as purging, laxative use, diet pills, or diuretics.

The patient’s vitals are within normal limits (Table 1). Her BMI is 30.6, and a mental status exam found her awake, alert, and oriented to person, place, and time (AAOx3), groomed, cooperative with "good" mood, clear speech, and full range and congruent affect. Her thought process was linear and organized and she denied auditory or visual hallucinations. Notably, she was found to have limited insight and judgement into her condition. A working diagnosis of unspecified eating disorder was assigned rather than anorexia nervosa, restricting type, as the latter remained a rule-out diagnosis at this initial evaluation pending further assessment. This distinction reflects a clinically cautious approach given her elevated BMI, which falls outside the traditional underweight threshold for anorexia nervosa. Although the Eating Disorder Examination Questionnaire (EDE-Q) was administered as part of the evaluation, it was not fully completed, highlighting the challenges of implementing standardized screening even when attempted in this population.

**Table 1:** Vital Signs and Anthropometric Measures for Four Patients With Atypical Eating Disorder Presentations Body mass index categories are based on World Health Organization definitions. Vital sign reference ranges reflect standard adult clinical norms. Values listed as "Not obtained" indicate measurements that were not collected at the time of initial psychiatric evaluation. Orthostatic vital signs were not obtainable across any of the four cases, as evaluations were conducted in an outpatient psychiatry setting without the nursing staff or medical equipment necessary to perform such measurements.

	Age	Sex Assigned at Birth	Gender Identity	Weight	BMI	Heart Rate	Blood Pressure	Temperature
Reference	-	-	-	-	18.5 - 24.9	60-100 beats per minute (bpm)	<120/80 mmHg	36.1-37.2 ℃
Case 1	17	Female	Female	78.4 kg	30.6 kg/m2	65 bpm	118/64 mmHg	36.6 ℃
Case 2	31	Female	Female	38.6 kg	16.6 kg/m2	70 bpm	Not Obtained	Not Obtained
Case 3	29	Female	Questioning	67.7 kg	22.0 kg/m2	68 bpm	125/82 mmHg	36.9 ℃
Case 4	18	Female	Female	58.1 kg	19.0 kg/m2	95 bpm	107/85 mmHg	36.3 ℃

Case 2

A 31-year-old English-speaking female with past psychiatric history of alcohol use disorder (AUD), generalized anxiety disorder, insomnia, and recurrent major depressive disorder (MDD) currently in remission presented to outpatient psychiatry clinic for evaluation of AUD. She had sought out an addiction specialist specifically for this concern, and disordered eating was not her stated reason for seeking care. Her history of disordered eating dated back to her sophomore year of high school, when she first began restricting her intake and engaging in compensatory exercise. Despite being followed by providers since 2017, no validated screening measures were employed, binge-purge behaviors were never documented or explored, and the connection between her alcohol use and eating disorder disinhibition went unrecognized until her 2023 addiction psychiatry evaluation. 

She reports drinking more than seven alcoholic beverages each night on weekends, feels she lacks control of her drinking, and has tried to quit or reduce use multiple times unsuccessfully. She engages in risky behaviors with her alcohol use that have resulted in legal consequences. Her friends have expressed concern about her use, and she endorses ongoing guilt about it. Further inquiry into her feelings of guilt revealed extreme distress with food bingeing episodes associated with intoxication. She is not preoccupied about her alcohol use per se, but more fixated on how her use enables her to be disinhibited with her food intake. Ongoing buildup of guilt for her intake afterwards leads to purging. When not intoxicated, she engages in restricted eating. Other triggers for binge eating behavior include zolpidem, prescribed by a previous provider for insomnia; however, its potential role in disinhibiting eating behaviors had not been explored prior to this evaluation. In the absence of known triggers, the patient reports restricting eating behavior. She reports rigidity in her eating practices, ongoing distress with her body image and weight, and a desire to achieve further weight loss.

Patient vitals were normal at the time of initial evaluation and BMI was 16.6 (Table 1). Mental status exam found her to be AAOx3, groomed, cooperative with "ok" mood, clear speech, and full range and congruent affect. Her thought process was organized, and she denied auditory or visual hallucinations. She had limited insight and judgement into her condition. A diagnosis of anorexia nervosa, binge/purge type, was established based on DSM-5, Text Revision (TR) criteria, including persistent restriction of energy intake, intense fear of weight gain, and recurrent compensatory purging behaviors. The initial presentation for AUD rather than eating concerns, combined with years of provider contact without comprehensive eating disorder screening, contributed to the significantly delayed recognition and treatment of the full extent of this patient's eating disorder. The EDE-Q 6.0 was administered as part of the evaluation, though completion was delayed. Results revealed clinically significant scores across all subscales, including marked restraint, eating concern, shape concern, and weight concern, consistent with the severity of her presentation and supportive of the diagnosis of anorexia nervosa, binge/purge type.

Case 3

A 29-year-old White English-speaking female with past psychiatric history of MDD, generalized anxiety disorder, attention-deficit/hyperactivity disorder (ADHD), and gender dysphoria presented to outpatient psychiatry for evaluation and management of ADHD, having been diagnosed by her primary care provider in 2021. The patient reported a longstanding history of disordered eating dating back to age 13. Manifestations included restrictive eating patterns, bingeing, purging, and excessive exercise. Exercise has since been limited by a medical comorbid condition. Despite having engaged with a nutritionist during high school and again in her early 20s, as well as a therapist, her eating disorder had persisted for over 15 years at the time of this evaluation. Currently, the patient reports eating one meal per day with light snacks. Purging occurs one time per week with no bingeing. Patient reports a history of competitive dance, which is stated to be a contributing factor due to body ideals in the sport at that time. Her eating behaviors became further restrictive as she started exploring her gender identity. She reports feeling that her sex does not align with her gender. She continues to use female pronouns and has adopted a gender-neutral name. Currently, she reports ongoing exploration of her gender identity and reports discomfort with her chest, specifically the presence of large breasts. Despite years of provider contact, she had never disclosed her eating behaviors, and no prior provider had screened for the potential relationship between her gender dysphoria and disordered eating. As her preoccupation and distress regarding her breasts worsened in the past, she began restricting further with increased compensatory exercise in attempts to lose weight and reduce the size of her breasts. These behaviors have ebbed and flowed over the years, although they have been worsening again lately.

Vitals were within normal limits; as noted in Table 1, her BMI of 22.0 kg/m² falls within the normal range. Mental status exam found the patient to be AAOx3, calm, and cooperative with normal speech and thought process. Her mood was found to be sad and anxious with congruent affect. She demonstrated good attention with fair insight and judgement into her condition. A diagnosis of unspecified feeding or eating disorder was assigned at this evaluation given the diagnostic complexity introduced by the intersection of gender dysphoria and disordered eating behaviors. While the clinical picture shares features with atypical anorexia nervosa under the OSFED framework, the unspecified diagnosis was intentionally selected to reflect the ongoing nature of the evaluation and the need for further assessment before a more specific classification could be made with confidence. Although the EDE-Q was administered as part of the evaluation, it was not completed and the patient was subsequently lost to follow-up, further illustrating the challenges of engaging this population in standardized screening and consistent care.

Case 4

An 18-year-old White Hispanic English-speaking female with past psychiatric history of MDD currently in full remission, borderline personality disorder (BPD), and unspecified anxiety disorder presented to the outpatient psychiatric clinic due to her mother's concerns for mood and disordered eating in the setting of changing environmental stressors. Patient states that her eating habits revolve around guilt and worry of consequences of her meal on her body habitus. Several times a month, the patient will binge, feel guilty, and purge. She currently eats two meals a day. She revealed how she initially engaged in these eating behaviors around age 10, when the family moved to South America. It was her first time living in a Latin American country. She did not have a say in the move and felt that many things in her life were changing without having a sense of control over her environment. Decisions were being made for her regarding the move and where she would study. She was leaving her friends behind and was faced with trying to assimilate into a new culture. She reports having had a lack of control and turned to changes in eating behaviors as her way of coping with these changes, and as a means of controlling something in her life. Her binge and purging was further perpetuated by a desire to be thinner and dissatisfaction with her body image as she was comparing herself to new cultural and beauty norms. Despite being followed by psychiatry at that time, her eating behaviors were not identified or targeted, representing a missed opportunity for early intervention. 

Patient vitals were normal and BMI was 19, as noted in Table 1. Mental status exam found that the patient was AAOx3 and cooperative with poor eye contact. Her speech was normal and her mood was "good" with congruent affect. The patient's thought process was linear and organized, with no auditory or visual hallucinations or suicidal ideation. The patient's insight and judgement into her medical condition was fair. A diagnosis of unspecified eating disorder was assigned, reflecting the complexity of disentangling primary disordered eating from behavioral responses to environmental stressors in the context of BPD. Although the EDE-Q was provided to the patient during the evaluation, it was not returned, consistent with the broader pattern of incomplete standardized screening observed across this case series.

## Discussion

Atypical presentations of eating disorders contribute to provider confusion and delays in symptom recognition [1,10]. A unifying pattern across all four cases presented in this series is that the eating disorder was not the primary reason for clinical contact. The patients presented for anxiety and trauma, alcohol use disorder, ADHD, and environmental stressors, respectively, and disordered eating was identified only through deeper inquiry or incidental disclosure. Prior provider contacts had occurred in each case without formal eating disorder screening, reflecting systemic gaps in clinical practice. It is important to acknowledge that some of this delay may also reflect disparities in healthcare access, as underrepresented populations face greater structural barriers to eating disorder identification and treatment [5]. Validated screening tools such as the Sick, Control, One, Fat, Food (SCOFF) questionnaire or the EDE-Q were administered across cases; however, completion was inconsistent, highlighting the challenges of implementing standardized screening even when attempted in this population. Furthermore, orthostatic vital signs were not obtainable across any of the four cases, as this series was conducted in an outpatient psychiatry setting without nursing staff or medical equipment necessary to perform such measurements. This represents an important systemic limitation and underscores the need for interdisciplinary collaboration between psychiatry and primary care in the management of eating disorder patients, particularly for medical monitoring.

Athletes, particularly those in aesthetic sports (e.g., figure skating, gymnastics, dance) or weight-class sports (e.g., powerlifting, wrestling), have higher rates of eating disorders than their peers in other sports [3,13]. The patient in Case 1 had a BMI of 30.6, which may have led providers to overlook an eating disorder diagnosis, as eating disorders can occur across a wide range of body sizes [1,10]. Additionally, the normalization of intense high school athletics may reduce provider concern regarding high-risk exercise behaviors [3]. Regardless of BMI, any rapid weight loss should prompt consideration of disordered eating [1]. Athletes should be considered for screening comprehensively, as they may engage in restrictive eating while maintaining body types traditionally considered "low risk" [3,13].

Trauma history is another critical factor in identifying eating disorders. Emotional abuse, physical neglect, and sexual trauma are well-documented risk factors for these conditions [14]. Case 1's extensive trauma history underscores the importance of routine trauma screening in both primary care and psychiatric settings to ensure appropriate intervention [14].

AUD can further mask atypical eating disorder presentations. Studies show a strong link between alcohol abuse and disorders such as anorexia nervosa and bulimia nervosa [15,16]. Providers often attribute weight loss in patients with AUD solely to alcohol use, overlooking coexisting eating disorders [15]. Additionally, individuals with an underweight BMI who consume alcohol have a higher all-cause mortality rate than their normal-weight peers [17]. Genetic correlations exist between alcohol dependence, binge eating, and compensatory behaviors, as seen in Case 2 [16]. Patients with chronic alcohol dependence, especially those experiencing weight fluctuations, should be screened for disordered eating to facilitate appropriate treatment [15,16].

Gender dysphoria also predisposes individuals to disordered eating and creates unique treatment challenges [18]. Studies indicate that 10.5% of transgender men and 8.1% of transgender women meet criteria for anorexia nervosa or bulimia nervosa [18]. Transmasculine individuals may restrict intake to minimize curves, while transfeminine individuals may restrict intake to achieve a smaller frame and alleviate dysphoria [18]. Weight gain during treatment may exacerbate gender dysphoria, increasing relapse risk [18]. Integrating gender-affirming care, including social transition, hormone therapy, or surgery, is crucial in managing eating disorders within this population [18].

Finally, identifying the underlying cause of disordered eating behaviors is essential for effective treatment [1,10]. In Case 4, the patient's disordered eating stemmed from a desire for control amid significant environmental stressors rather than a drive for thinness. BPD further increases the patient's risk, as individuals with BPD often engage in disordered eating as a response to rejection or loss of control [19]. In such cases, dialectical behavioral therapy, alongside medication management, should target the root cause to achieve long-term remission [19].

## Conclusions

Atypical presentations of eating disorders often diverge from classical diagnostic profiles, contributing to provider confusion, misdiagnosis, and delayed treatment. This case series highlights how factors such as athletic participation, trauma history, substance use disorder, gender dysphoria, and environmental stressors can obscure recognition of disordered eating, particularly in populations not traditionally perceived as high risk. Clinicians should maintain a high index of suspicion for eating disorders regardless of BMI or presenting complaint and broaden screening efforts to include underrecognized at-risk groups. Current screening protocols such as the EDE-Q represent valuable tools for identifying disordered eating across atypical presentations; however, as demonstrated in this series, completion of such measures can be challenging even when administered, underscoring the need for improved screening infrastructure and patient engagement strategies in outpatient psychiatric settings. Improved awareness of these nonclassical presentations, along with comprehensive psychosocial assessment and routine interdisciplinary collaboration between psychiatry and primary care, may facilitate earlier identification, timely intervention, and improved patient outcomes.
